# Post-Translational Modifications of Transcription Factors Harnessing the Etiology and Pathophysiology in Colonic Diseases

**DOI:** 10.3390/ijms21093207

**Published:** 2020-05-01

**Authors:** Chao-Yuan Hsu, Shin-Huei Fu, Ming-Wei Chien, Yu-Wen Liu, Shyi-Jou Chen, Huey-Kang Sytwu

**Affiliations:** 1 National Institute of Infectious Diseases and Vaccinology, National Health Research Institutes, No.35, Keyan Road, Zhunan, Miaoli 350, Taiwan; hsu.chaoyuan@gmail.com (C.-Y.H.); winniefold@gmail.com (S.-H.F.); 2 Department and Graduate Institute of Microbiology and Immunology, National Defense Medical Center, No.161, Section 6, Min Chuan East Road, Neihu, Taipei 114, Taiwan; pantherchien@gmail.com (M.-W.C.); pedneuchen@hotmail.com (S.-J.C.); 3 Graduate Institute of Life Sciences, National Defense Medical Center, No.161, Section 6, Min Chuan East Road, Neihu, Taipei 114, Taiwan; candy_77615@yahoo.com.tw; 4 Molecular Cell Biology, Taiwan International Graduate Program, No.128, Academia Road, Section 2, Nankang, Taipei 115, Taiwan; 5 Department of Pediatrics, Tri-Service General Hospital, National Defense Medical Center, No. 325, Section 2, Chenggong Rd., Neihu District, Taipei 114, Taiwan

**Keywords:** transcription factor, phosphorylation, methylation, acetylation, ubiquitination, SUMOylation, O-GlcNAcylation

## Abstract

Defects in mucosal immune balance can lead to colonic diseases such as inflammatory bowel diseases and colorectal cancer. With the advancement of understanding for the immunological and molecular basis of colonic disease, therapies targeting transcription factors have become a potential approach for the treatment of colonic disease. To date, the biomedical significance of unique post-translational modifications on transcription factors has been identified, including phosphorylation, methylation, acetylation, ubiquitination, SUMOylation, and O-GlcNAcylation. This review focuses on our current understanding and the emerging evidence of how post-translational regulations modify transcription factors involved in the etiology and pathophysiology of colonic disease as well as the implications of these findings for new therapeutic approaches in these disorders.

## 1. Introduction

Colonic diseases comprise a wide range of intestinal diseases, including inflammatory bowel disease (IBD), colon cancer, and diverticular disease. IBD, which includes ulcerative colitis (UC) and Crohn’s disease (CD), is regarded as a global problem because its prevalence is increasing in North America, Europe, and Asia, whereas its incidence is accelerating in newly industrialized countries [1,2]. IBD is a chronic inflammatory disorder of the intestine characterized by phases of remission and relapse. Assessment of disease distribution in the colon by endoscopic biopsy is helpful to distinguish UC from CD. UC is characterized by superficial inflammation that is limited to the mucosa and submucosa of the colon, whereas CD is characterized by transmural inflammation that affects all layers of mucous membrane forming the gastrointestinal wall [3]. Moreover, chronic inflammation in intestinal tissues is a major driving force for the initiation and progression of tumors, whereas tumor-infiltrating lymphocytes that produce tumor-promoting cytokines are thought to make a major contribution to the development of colon cancer [4]. Previous studies have linked disruption of the epithelial barrier to the increase of pro-inflammatory cytokines in the gut that eventually contribute to the development of IBD, which also predispose patients to the development of colitis-associated colon cancer (CAC) [5,6]. The integrity and barrier properties of epithelial layers are regulated by tight junction and adherens junction. Augmented expression of mucosal pro-inflammatory cytokines during the intestinal inflammation causes a disturbance in the intestinal junctional barrier to promote the transepithelial permeability and increases tissue penetration of luminal antigens [7]. It has been reported that pro-inflammatory cytokines such as interferon-γ(IFN-γ) and tumor necrosis factor-α (TNF-α) negatively regulate the barrier properties and self-renewal of the intestinal epithelium to exacerbate mucosal inflammation [8,9]. On the contrary, IL-6- and IL-22-mediated signaling pathways in the intestinal epithelium have been reported to modulate the proliferation of epithelial cells during the development of experimental colitis [10,11]. These results indicated that pro-inflammatory cytokines serve as important regulators in maintaining epithelial homeostasis and the mucosal barrier in the intestine. Moreover, emergency surgery is needed for IBD patients when they are suffering from life-threatening complications, such as uncontrolled bleeding occurring from deep ulcerations, toxic megacolon, perforation of the colon, and the presence of fistula or abdominal abscess, that contribute to an increased mortality [12]. Surgery is considered as a cure for UC and as an approach to improve the symptoms and the quality life for CD patients. Approximately 20% of patients with UC will require surgery, whereas up to 80% of patients with CD will undergo an operation during their lifetime. The standard of care in the treatment of UC requiring surgery is laparoscopic colectomy and restorative ileoanal pouch anastomosis (IPAA), whilst laparoscopic ileo-cecal resection is already the new gold standard in the treatment of complicated CD of the terminal ileum [13]. Thus, many patients with IBD-related complications have to undergo surgery, which highlights the requirement to develop new therapeutic strategies for colonic diseases.

The development of IBD is markedly influenced by an individual’s genetic predisposition, aberrant immune responses, or environmentally associated dysregulation of the gut microbiota. Further evidence of a critical role for cytokines in the pathogenesis of IBD has been noted in genetic studies. Genome-wide association studies (GWAS) have detected single-nucleotide polymorphisms (SNPs) in genes encoding cytokines or transcription factors that control cytokine-mediated activation of immune cells associated with the pathogenesis of IBD [14,15]. The aberrant immune response toward the commensal microbiota is responsible for disease in genetically susceptible individuals [16]. Current evidence from animal models of colitis and human studies reveal that both innate lymphoid cells (ILCs) and CD4^+^ T helper (Th) cells are major mediators for the pathogenesis of chronic intestinal inflammation in IBD [17,18]. ILCs belong to a family of innate immune cells that share similarities with the phenotype and functions of T lymphocytes. They are considered to function as key orchestrators of immune defenses at mucosal surfaces and to be crucial for maintaining an intact intestinal barrier [17]. Intestinal ILCs are compartment specific, with ILC1 representing the major fraction in the upper gastrointestinal tract and ILC3 signifying the major population in ileum and colon [19]. ILC2 cells are present at very low frequencies over the entire intestine, but they have been reported to play critical roles in mounting protective innate responses against parasites and helminths [20,21]. In addition, CD4^+^ T cells under TCR activation and cytokine stimulation can differentiate into several T helper cell lineages, which indicate that naïve T cells differentiate into distinct effector subsets, such as T helper 1 (Th1), Th2, Th17, and regulatory T cells, in response to various pathogens and play an important role in the immune system [18,22]. The functions and signaling requirements for development of ILCs and T helper cells are dependent on the networks of transcription factors involved in their differentiation [17,18]. Post-translational modifications (PTMs) are important in modulating the activity and stability of transcription factors (TFs) by forming covalent linkages with the functional groups of TFs. The advantage of PTMs is that they can dynamically regulate transcription factors at a much faster rate and with a lower energy cost than protein turnover. Generally, protein PTMs can rapidly modulate complex formation, stability, activity, and spatial localization, including phosphorylation, acetylation, methylation, ubiquitination, SUMOylation, and O-GlcNAcylation. Protein phosphorylation catalyzed by kinases and phosphatases at the serine, threonine, or tyrosine resides of transcription factors is a reversible and transient modification and is important for facilitating the recruitment of co-activators to enhance transcription activation. The phosphorylation of target proteins during T cell activation is involved in protein intracellular stability, interaction, and localization and triggers a signaling cascade with rapid phosphorylation events to reprogram the proteomes and bioenergetic features [23].

Protein acetylation catalyzed by histone acetyltransferases (HATs) and histone/protein deacetylases (HDACs) at the lysine or methionine residue is a reversible process that modulates a number of functional properties of transcription factors [24]. Protein acetylation is a major regulator of gene transcription since most of the canonical HATs localize in the nucleus and function as transcription co-activators to modulate the sequence-specific DNA binding activity and cellular metabolism in response to internal or external stimulation for immune cells [25]. Consistent with phosphorylation and acetylation, protein methylation can be monomethylated, dimethylated or trimethylated at lysine by lysine methyltransferase (KMT) or monomethylated, asymmetrical dimethylated or symmetrical dimethylated at arginine residues by protein arginine methyltransferase (PRMT) [26]. The effects of methylation on protein–protein and protein–DNA interactions, protein subcellular localization, and protein stability can modulate many cellular processes [27].

Protein ubiquitination, inversely regulated by E1, E2, E3 enzymes and deubiquitylation enzymes (DUBs), plays important roles in a wide variety of immunological processes such as cell differentiation and immune defense. Lys48-linked ubiquitination promotes the proteasomal degradation of targeted proteins, whereas Lys63-linked ubiquitination is involved in the transduction of signaling pathways [28]. Similar to the ubiquitination cascade, the conjugation of the small ubiquitin-like modifier (SUMO) to specific lysine residues of cellular proteins (SUMOylation) is a dynamic and reversible posttranslational modification by a specific enzyme cascade, including SAE1/SAE2 (E1-activating enzymes), UBC9 (E2-conjugating enzyme), and multiple E3 ligases [29,30]. Nuclear proteins such as transcription factors are one of major groups among SUMOylation substrates, while SUMOylation can regulate many cellular processes, including nuclear transport, transcription, chromosome segregation, and DNA repair [31].

The modification of protein O-GlcNAcylation is a non-canonical glycosylation that involves the attachment of single O-linked N-acetylglucosamine (O-GlcNAc) moieties to serine and threonine residues of transcription factors and is catalyzed by O-GlcNAc transferase (OGT) and O-GlcNAcase (OGA) [32]. O-GlcNAcylation has been proposed to regulate genetic transcription and cellular metabolism to serve as a nutrient and stress sensor that is highly sensitive to changes in the cellular environment [33]. Moreover, O-GlcNAcylation has been shown to occur reciprocally or sequentially with phosphorylation on the associated residues of target proteins [34]. This review focuses on our current understanding and the emerging evidence of how post-translational regulations modify transcription factors involved in the etiology and pathophysiology of colonic disease as well as the implications of these findings for new therapeutic approaches in these disorders.

## 2. Overview of Post-Translational Modifications of Transcription Factors and the Modulatory Effects on Colonic Disease

ILC and T helper cells are key in mediating the host protective and homeostatic responses, whereas they are also known to be the main drivers of IBD. In this part, we focus on the critical roles of PTMs in the positive or negative modulation of transcription factor-based cytokine regulation by targeting ILCs and CD4^+^ T cell differentiation towards inflammatory phenotypes and its implications for pathogenesis of the colonic diseases.

### 2.1. T-bet

The T box transcription factor (T-bet), which is encoded by *Tbx21*, plays crucial roles in the pathophysiology of experimental colitis and CD via regulation of the genetic programs of multiple lineages of immune cells such as ILC1 and Th1 cells to modulate the balance of interleukin (IL)-12- or IL-23-driven mucosal cytokine production [35,36,37]. Overexpression of T-bet in CD4^+^ T cells exacerbated experimental colitis, whereas *Tbx21*-deficient CD4^+^ T cells were unable to modulate the development of colitis [36]. Interestingly, T-bet can also play a protective role in murine IBD. T-bet-knockout mice exhibit more severe colitis than controls during the development of DSS-induced colitis [38]. ILC1s that express T-bet modulate the defense against *Helicobacter typhlonius* and mice with T-bet-deficient innate immune cells spontaneously develop *H. typhlonius*-mediated intestinal inflammation because of dendritic cell-restricted tumor necrosis factor (TNF)-α production [21].

T-bet is a critical modulator of type 1 inflammatory responses and the levels of T-bet phosphorylation have been linked with the activation of host immune defense responses against infectious microorganisms during the development of intestinal inflammation. Tyr525-linked phosphorylation of T-bet interferes with the binding of Th2 cell-associated transcription factor Gata-binding protein-3 (Gata3) to its target DNA [39]. Glycogen synthase kinase 3 (GSK3)-modulated T-bet phosphorylation at Ser508 impairs RelA-mediated *Il2* transactivation [40]. Thr302-based phosphorylation of T-bet is critical for its interaction with NFAT1, a deficiency of which inhibits the ability to suppress NFAT1-mediated regulation of cytokine production [41]. Moreover, phosphorylation of Ser498 and Ser502 of T-bet was required for the inhibition of colon cancer metastasis and growth via positive regulation of RSK2/T-bet/interferon (IFN)-γ signaling [42]. T-bet with constitutive phosphorylation is able to restore the IFN-γ mRNA levels and dramatically reduced the rate of colon cancer liver metastasis in mice [42], suggesting that phosphorylation positively modulates T-bet-based IFN-γ production to regulate the colon cancer metastasis. Lys313-linked ubiquitination of T-bet also modulates its phosphorylation at Thr302 and hence its degradation, and affects functions involving DNA binding and transcriptional activation of IFN-γ [41]. Mass-spectrometry proteomic analysis revealed that mTORC1 can also promote T-bet phosphorylation to regulate Th1 differentiation [43]. Although single-phosphorylation-site mutants still support induction of IFN-γ expression, simultaneous mutation of three of the mTORC1-dependent sites results in significantly reduced IFN-γ production. The reduced activity of the triple mutant T-bet is associated with its failure to recruit chromatin-remodeling complexes to the *Ifng* gene promoter [43]. In addition, c-Abl-mediated triple phosphorylation of T-bet at Tyr219/Tyr265/Tyr304 regulates its ability to bind to the DNA sequences of its target genes and hence modulates gene expression [44], and Tyr304-based phosphorylation of T-bet is required for formation of the T-bet–Runx1 complex that suppresses development of the Th17 cell lineage by inhibiting transcription of *Rorc*, which encodes the transcription factor of retinoic acid-related orphan receptor (RORγt) [45]. These studies suggested that the transactivation ability of T-bet is specifically regulated by phosphorylation and ubiquitination.

### 2.2. Gata3

The transcription factor Gata3 has emerged as an important regulator of both innate and adaptive immunity. Gata3 belongs to the GATA-binding protein family and has conventionally been regarded as a master transcription factor for the differentiation of Th2 by directly transactivating the *Il4, Il5,* or *Il13* genes. Gata3 also plays critical roles in promoting the production of IL-5 and IL-33 in ILC2 cells, and regulates IL-9 production in Th9 cells. It has been reported that the expression levels of GATA3 mRNA were increased in both pediatric and adult patients with UC and that high levels of protein were expressed in CD4^+^ T cells from the lamina propria of patients with UC [46,47]. Moreover, the mucosal expression of GATA3 was positively associated with disease activity in adult patients with UC and correlated with the production of inflammatory cytokines in both patients with UC and in models of experimental colitis [47]. A recent detailed analysis of the T-cell subsets involved in the development of IBD revealed that IL-9-producing Th9 cells expressing the transcription factors GATA3 and PU.1 were more frequently observed in the mucosa of patients with UC than in that of patients with CD [48,49]. Moreover, it was reported that patients with UC that had increased serum levels of IL-9 had a worse prognosis and that IL-9 production was correlated with their disease status [50,51]. Genetic ablation of *Gata3* in mouse T cells was shown to contribute to significant inhibition of IL-9 expression in oxazolone-induced colitis [47]. Therefore, Gata3 plays critical roles in modulating multiple lineages during the development of intestinal inflammation.

It has been reported that Arg261-based methylation of the N-finger domain of Gata3 is critical for its regulation of heat shock protein 60 (Hsp60)-associated negative regulation of *Il5* gene expression in Th2 cells, suggesting that arginine methylation plays a pivotal role in the organization of Gata3 complexes and their target gene specificity [52]. Akt1-mediated phosphorylation of Gata3 at Ser308, Thr315, and Ser316 represses T-bet-mediated and memory Th2 cell-restricted IFN-γ production by inducing the dissociation of histone deacetylase 2 (HDAC2) from the Gata3/Chd4 repressive complex [53]. In ILC2 cells, p38-mediated phosphorylation of Gata3 regulates the production of IL-6 by ILC2 [54]. It has also been reported that Gata3 associates with SUMO-E2 conjugating enzyme UBC9 and the SUMO-E3 ligase PIAS1 in yeast two-hybrid assays [55]. Overexpression of PIAS1 enhances Gata3 binding to the *Il13* promoter and enhances IL-13 production in splenocytes, whereas PIAS1 has a minimal enhancing effect on Gata3 binding to the *Il4* promoter to promote IL-4 production [55]. Taken together, these results suggest that the phosphorylation-, methylation- and SUMOylation-mediated modifications are important for the regulation of Gata3 in immune cells.

### 2.3. RORγt

The RORγt is a key transcription factor involved in Th17 cell differentiation through direct transcriptional activation of IL-17. The ILC3 cells that participate in the response against extracellular pathogens at mucosal sites also depend on expression of RORγt and, like their Th17 counterparts, secrete IL-17, IL-22, GM-CSF, and TNF-α [16]. RORγt-expressing ILC3 cells are known to play critical roles in the development of colitis because the intestinal inflammation was abrogated in mice lacking ILC3 cells [56]. Importantly, E3 ubiquitin ligase Itch-mediated ubiquitination of RORγt for proteasomal degradation was recently shown to limit IL-17 production. Mice deficient for Itch developed spontaneous colitis at 6–8 months of age associated with increased IL-17 levels in mucosal tissues and also exhibited higher tumor burden and increased incidence of colonic inflammation-associated cancer [57], indicating that Itch-modulated and RORγt-based ubiquitination can regulate IL-17-mediated colonic inflammation-associated cancer. The E3 ligase UBR5, which belongs to the UBR-box family, also interacts with RORγt in Th17 cells. Genetic ablation of UBR5 in Th17 cells can promote the stability of RORγt protein and enhance IL-17 production [58]. Furthermore, TRAF5-mediated Lys63-linked polyubiquitination plays an essential role in the positive regulation of IL-17 expression by RORγt [59]. Several deubiquitinases have been reported to regulate Th17 cell differentiation. The deubiquitinase DUBA, is a negative regulator of IL-17 production in T cells via stabilization of UBR5 to downregulate the stability of RORγt and mice with DUBA-deficient T cells exhibited exacerbated inflammation in the small intestine after challenge with anti-CD3 antibodies [58]. The E3 deubiquitinase USP4, interacts with and deubiquitinates Lys48-linked polyubiquitination of RORγt to enhance RORγt-mediated IL-17A transcription [60]. Moreover, USP17 is a positive regulator of RORγt in Th17 cells, whereas USP18 has been reported to modulate T cell activation and Th17 cell differentiation by deubiquitinating of the TAK1–TAB1 complex [61] and USP25 has been regarded as a negative regulator of IL-17-mediated inflammation via TRAF5 and TRAF6 deubiquitination [62].

**Figure 1:**
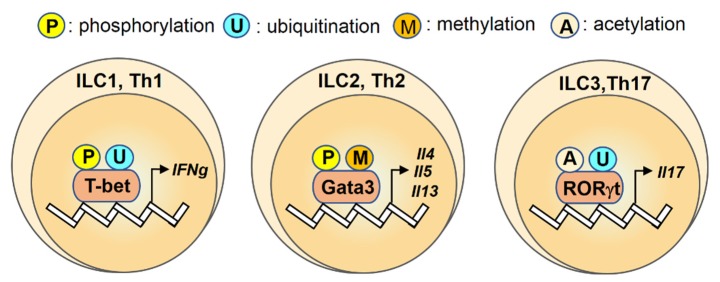
An overview of regulation for transcription factors T-bet, Gata3, RORγt by post-translational modifications (PTMs) in innate lymphoid cells (ILCs) and T helper cells. Transcription factors T-bet, Gata3 and RORγt are regulated by the post-translational modifications (P: phosphorylation; U: ubiquitination; M: methylation; A: acetylation) and the color differences indicate various modifications on their targets.

Acetylation of transcription factors often competes for the same lysine residues as ubiquitinylation. Acetylation at these sites protects these transcription factors from ubiquitin-mediated proteasomal degradation. Mass spectrometry and mutation studies reported that histone acetyltransferase p300 can mediate acetylation of the DNA-binding domain of RORγt at the Lys69, Lys81, Lys99, and Lys112 residues [63]. The deacetylase Sirtuin 1 (SIRT1) enhances the transcriptional activity of RORγt to promote the function of Th17 cells, whereas genetic ablation or pharmacologic inhibition of Sirt1 suppresses Th17 differentiation [63]. Moreover, the p300-mediated acetylation of RORγt at Lys81 residue promotes RORγt-associated gene expression in Th17 cells, whereas HDAC1 reduces its acetylation levels and the transcriptional activation of IL-17 [64]. Taken together, these studies suggest that the transactivation ability of RORγt is specifically regulated by ubiquitination and acetylation (Figure 1).

### 2.4. STAT3

STAT3 is activated via phosphorylation of the Tyr705 and Ser727 residues by kinases associated with the IL-6 and IL-21 receptors and is critical for in vitro and in vivo differentiation of Th17 cells [65]. It has been reported that the phosphorylation level of STAT3 is enhanced in intestinal T cells from patients with CD and is positively correlated with disease severity. GWAS have revealed that a *STAT3* SNP is associated with enhanced IBD susceptibility or disease severity in different populations, further implicating a role for STAT3 in both CD and UC. Stat3 signaling also plays critical roles in a mouse model of IBD, i.e., genetic ablation of *Stat3* in CD4^+^ T cells contributed to the defect of Th17 cell differentiation and then were unable to induce intestinal inflammation during the development of T cell-mediated colitis, suggesting a critical role for T cell-intrinsic Stat3 in the progression of colitis. Moreover, STAT3 plays important roles in the regulation of signaling between cytokines and their receptors in the pathway to colitis [65]. Previous studies reported that the modification of O-GlcNAcylation of Stat3 at the Thr717 residue suppresses its phosphorylation and the expression of the downstream anti-inflammatory cytokine IL-10 and contributes to the augmented disease severity in azoxymethane (AOM)-induced colitis and colitis-associated cancer, whereas myeloid-derived cullin 3 promotes Stat3 phosphorylation by inhibiting OGT expression and protects against intestinal inflammation [66]. These results indicate that both phosphorylation and O-GlcNAcylation are critical for the regulation of Stat3 in T cells.

### 2.5. IRF4

IFN regulatory factor 4 (IRF4), a member of the IRF family of transcriptional regulators, acts as a critical regulator for the development of mucosal T cell subsets, including Th9, Th17, and Treg cells [49,67,68,69]. Patients with either CD or UC exhibited augmented IRF4 expression in lamina propria T cells compared with control patients [66]. The expression of IRF4 mRNA and the number of IRF4^+^ cells were significantly increased in patients with UC and positively correlated with disease severity [49]. In murine models, genetic ablation of *Irf4* protected mice from experimental colitis because of decreased production of IL-6 [67,70]. It has been reported that serine-threonine kinase ROCK2-mediated phosphorylation of IRF4 regulate the differentiation of Th17 cells through modulation of the production of IL-17 and IL-21, and that administration of ROCK inhibitors ameliorates IL-17- and IL-21-mediated inflammatory responses in mice [71]. IRF4 also cooperates with Foxp3 in Treg cells to control the Th2 response [72]. UBC9-mediated SUMOylation of IRF4 at Lys349 is critical for promoting its protein stability and its ability to regulate T-cell receptor-dependent gene expression in Treg cells [73]. These results imply that both phosphorylation and SUMOylation are critical for the regulation of IRF4 in T cells.

### 2.6. c-Maf

The transcription factor c-Maf, which belongs to the AP-1 family of basic-region and leucine-zipper transcription factors, was first reported as a Th2-specific gene that enhance *Il4* transactivation through direct binding to the consensus *Maf* recognition element in the proximal *Il4* promoter, but not the *Il5* or *Il13* promoters [74]. It has been reported that overexpression of c-Maf in naïve CD4^+^ T cells reduces Th1-mediated colitis compared with wild-type controls, but memory T cells with enhanced c-Maf expression exacerbate the development of colitis [75]. Moreover, the numbers of c-Maf-enriched T cells are increased in the inflamed intestine of patients with CD or UC and the Th1-speficic transcription factor T-bet is reported to be coexpressed with c-Maf in T cells [75]. In addition to its roles in Th2 cells, recent studies reported that c-Maf promotes IL-10 and/or IL-21 expression in Th17, Tr1, and T follicular helper (Tfh) cells [76,77,78], and c-Maf was also recently found to be required for the differentiation of microbiota-induced RORγt-expressing Treg cells in response to an intestinal pathobiont [79]. It also was regarded as a key regulator of intestinal Treg cell function that is instrumental in establishing and maintaining host–microbiota homeostasis. Treg cell-specific c-Maf deficiency resulted in microbial dysbiosis and an increase in intestinal IgA-producing plasma cells [80].

**Figure 2:**
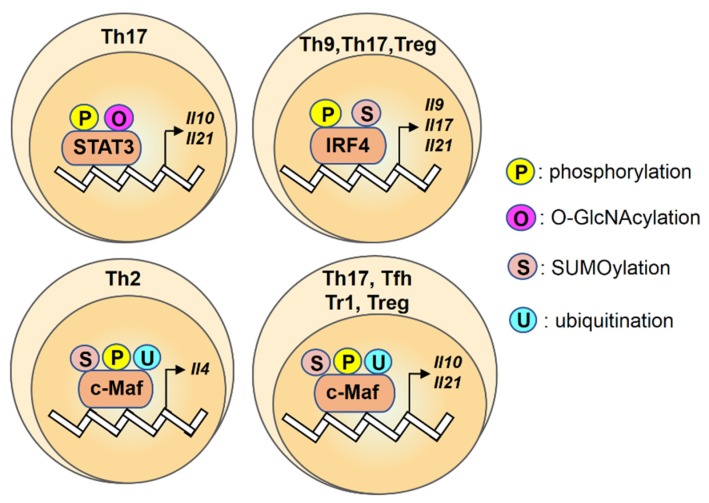
An overview of regulation for transcription factors STAT3, IRF4, c-Maf by post-translational modifications in T helper cells. Transcription factors STAT3, IRF4, c-Maf are regulated by the post-translational modifications (P: phosphorylation; O: O-GlcNAcylation; S: SUMOylation; U: ubiquitination) and the color differences indicate various modifications on their targets.

Post-translational phosphorylation, SUMOylation, and ubiquitination can regulate the function of c-Maf protein by modifying its activity, subcellular localization, and half-life. CARMA1/IKK-mediated phosphorylation of c-Maf promotes its DNA binding activity but does not affect c-Maf abundance [81]. Regulation of tyrosine phosphorylation of c-Maf by kinase TEC and the phosphatase Pep is also critical for its recruitment to the *Il4* and *Il21* promoters and for optimal cytokine production [82]. SUMOylation at the Lys33 residue of c-Maf can reduce its ability to bind the *Il4* promoter and decrease its transactivating activity in a luciferase reporter assay [83,84]. Moreover, the levels of UBC9- and PIAS1-mediated c-Maf SUMOylation are inversely correlated with the expression of *Il21* in CD4^+^ T cells [78]. T-cell specific overexpression of c-Maf with a mutated SUMOylation site inhibits Daxx/HDAC2 recruitment to the *Il21* promoter and enhances CREB-binding protein- and p300-mediated histone acetylation [78]. These results suggest that the SUMOylation status of c-Maf has a stronger regulatory effect on IL-21 than the level of c-Maf expression, probably through an epigenetic mechanism. It was also reported that any single lysine mutation of c-Maf is not able to prevent its ubiquitination, suggesting that ubiquitination of this transcription factor is mediated by multiple lysine residues [85]. Subsequent studies demonstrated that the ubiquitin-conjugating enzyme UBE2O can mediate ubiquitination of c-Maf at the Lys331 and Lys345 residues and the ubiquitin ligase HERC4 can modulate Lys85- and Lys297-linked ubiquitination of c-Maf [86,87]. Taken together, these studies suggest that the transactivation ability and protein stability of c-Maf is specifically regulated by post-translational phosphorylation, SUMOylation, and ubiquitination (Figure 2).

### 2.7. Blimp-1

GWAS have reported that the *PRDM1*, which encodes B lymphocyte-induced maturation protein-1 (BLIMP-1), is an IBD susceptibility gene [14,88]. Blimp-1 plays critical roles in the modulation of mucosal T cell subsets including Th17, Treg, and type 1 regulatory (Tr1) cells [68,89,90]. Blimp-1 is a transcriptional repressor that is required for the maintenance of T cell homeostasis because mice specifically lacking T-cell expression of Blimp-1 or mice reconstituted with Blimp-1-deficient fetal liver cells, accumulated activated T cells and developed immune pathology and colitis [91,92]. The colitogenic phenotype of Blimp-1 knockout mice was alleviated when an IL-23 knockdown transgene was introduced, indicating the therapeutic potential of downregulating the IL-23–Th17 axis in these knockout mice [89]. It has been reported that Blimp-1 suppresses genes by interacting with several chromatin-modifying enzymes such as HDAC1 and HDAC2 [93]. Previous studies revealed that SUMOylation of Blimp-1 regulates its intracellular stability [94]. SUMO E3 ligase PIAS1 can mediate SUMOylation of Blimp-1 at Lys816 and mutation at the Lys816 residue of Blimp-1 reduced transcriptional repression correlating with a reduced interaction with HDAC2 [95]. These results imply that SUMOylation play an important role in the regulation of Blimp-1.

### 2.8. Foxp3

The transcription factor Foxp3 is a master lineage regulator for the development and suppressive activity of Treg cells, which are required to maintain intestinal immune homeostasis and prevent the development of colonic inflammation. Accumulating evidence indicates that the activity of Foxp3 protein is modulated by various post-translational modifications, including phosphorylation, acetylation, ubiquitination, and O-GlcNAcylation. CDK2-mediated phosphorylation of Foxp3 at Ser19 and Thr175 negatively modulated its protein stability and transcriptional activity, hence downregulating the function of Treg cells to promote the development of colitis, suggesting that CDK2-mediated and Foxp3-based phosphorylation can modulate the intestinal inflammation [96,97]. Moreover, the expression levels and transcriptional activity of Treg cells also depend on the discrete phosphorylation of Foxp3 by serine/threonine kinases such as Pim-1 and Pim-2 [98,99]. Pim-1-modulated and Ser422-linked phosphorylation on the forkhead domain of human FOXP3 attenuate its DNA binding activity and contribute to the reduced expression of Treg cell-associated genetic profiles [98]. A deficiency of Pim-2 activity enhances the suppressive activity of Treg cells and promotes murine host resistance to the development of DSS-induced acute colitis [99]. These findings demonstrate that phosphorylation of Foxp3 by CDK2, Pim-1, and Pim-2 negatively regulates the transcriptional activity of Foxp3 to inhibit the suppressive function of Treg cells. In contrast, Ser418-based FOXP3 phosphorylation plays a positive role in regulating the Treg suppressive function [100]. Protein phosphatase 1-mediated dephosphorylation of FOXP3 at Ser418 limits Treg cell activity and contributes to enhancing the pathogenicity of Th1 and Th17 cells. Ser418 phosphorylation of FOXP3 also modulates the DNA binding affinity of Foxp3 by interactions with acetylation-associated proximal modification [101].

Treatment of mice with pan-HDAC inhibitors such as TsA and SAHA enhanced the formation and suppressive activity of Treg cells to prevent the development of DSS-induced or T cell-induced colitis [102]. Targeting certain HDACs, especially HDAC6, HDAC9, and Sirtuin-1, could enhance the suppressive activity of Treg cells via modulation of the levels of acetylated Foxp3 to affect its ubiquitination and proteasomal degradation [103]. A previous mass-spectrometry study of immunoprecipitated proteins identified that Foxp3 could be acetylated at the Lys31, Lys262, and Lys267, and that SIRT1-mediated deacetylation at these target sites of Foxp3 contributed to negative regulation of Treg function [104]. In addition, the protein stability and expression of Foxp3 are regulated by ubiquitin-specific protease (USP)-dependent deubiquitylation. USP7-modulated deubiquitylation promotes Treg suppression and deubiquitinase inhibitor-treated Treg cells shows augmented modifying functions in adoptive transfer-induced colitis [105,106]. In addition, USP21 prevents the depletion of FOXP3 protein by mediating its deubiquitination and thereby maintaining the expression of Treg signature genes and Treg-specific deficiency of Usp21 contributed to the development of Th1-type inflammation [107].

**Figure 3:**
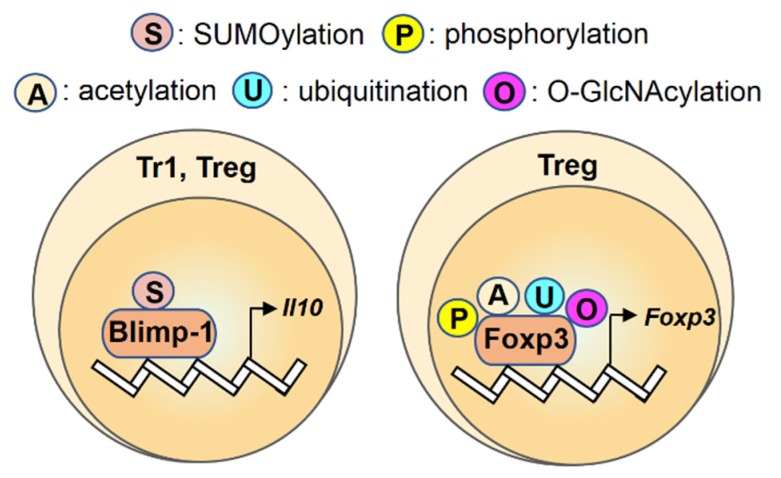
An overview of regulation for transcription factors Blimp-1 and Foxp3 by post-translational modifications in Tr1 and Treg cells. Transcription factors Blimp-1 and Foxp3 are regulated by the post-translational modifications (S: SUMOylation; P: phosphorylation; A: acetylation; U: ubiquitination; O: O-GlcNAcylation) and the color differences indicate various modifications on their targets.

Recent studies reported that levels of protein O-GlcNAcylation and the expression of O-GlcNAc transferase (OGT) were reduced in intestinal epithelial cells (IEC) of patients with UC and CD [108,109]. Similar to phosphorylation, OGT-modulated O-GlcNAcylation of Foxp3 is also critical for the lineage stability and effector function of Treg cells. Mice with OGT deficiency either in IEC or Treg cells exhibited severe intestinal inflammation, indicating that OGT-modulated and Foxp3-based O-GlcNAcylation can regulate the development of colitis [108,110]. Pharmacological enhancement of intracellular O-GlcNAc levels promoted the increase in SOCS3 gene expression and the suppressive activity of human Treg cells [110]. Taken together, these studies suggested that the transactivation ability and protein stability of Foxp3 are specifically modulated by multiple post-translational modifications, including phosphorylation, acetylation, ubiquitination and O-GlcNAcylation (Figure 3).

## 3. Conclusions

This review summarizes the findings that have led to a better understanding of the modulatory role of post-translational modifications (Table 1) on several transcription factors in ILC and T helper cells during the development of colonic disease (Figure 1, Figure 2 and Figure 3). Given the complexity of the function of post-translational modifications, future works that aim to clarify the effects of these protein modifications in colonic disease may focus on the following points: to analyze the profiles of the lineage-specific transcription factors with post-translational modifications in immune cells from different clinical samples systematically and to correlate these profiles with stages of colonic disease; to characterize T cell phenotypes of patients with CD or UC, especially for the colon-infiltrating lymphocytes; to explore the underlying mechanism of the altered post-translational modifications in colonic tissues. Taken together, studying post-translational modifications on transcription factors and identifying its associated immune disorders has revealed important aspects of this regulatory machinery and may help to provide new insights into the immune regulation as well as the development of therapeutic treatment targeting transcription factors with specific post-translational modifications rather than broad immunosuppressive agents.

## Figures and Tables

**Table 1:** The modulatory effects of PTMs on transcription factors.

Transcription Factor	Position	Modification	Physiological Effect	Ref.
T-bet	Tyr525	phosphorylation	inhibition of Gata3 binding ability and promotion of IFNγ-associated inflammation	[39]
	Ser508	phosphorylation	inhibition of IL-2 production	[40]
	Thr302	phosphorylation	interaction with NFAT1 and regulation of IFNγ-associated inflammation	[41]
	Ser498/Ser502	phosphorylation	promoting IFNγ production for inhibition of colon cancer metastasis	[42]
	Tyr219/Tyr265/Tyr304	phosphorylation	modulation of the binding ability to promote IFNγ-associated inflammation	[44]
	Tyr304	phosphorylation	inhibition of Th17 cell development and IL-17-associated inflammation	[45]
	Lys313	ubiquitination	regulation of T-bet protein stability and IFNγ-associated inflammation	[41]
Gata3	Arg261	methylation	regulation of IL-5 production to promote Th2 inflammation	[52]
	Ser308/Thr315/Ser316	phosphorylation	inhibition of T-bet-mediated and IFN-γ-associated inflammation	[53]
		phosphorylation	regulation of IL-6 production	[54]
		SUMOylation	promotion of Gata3 binding ability to enhance Th2 inflammation	[55]
RORγt		ubiquitination	regulation of RORγt protein stability for inhibition of Th17 inflammation	[57,58]
	Lys63	ubiquitination	regulation of IL-17 production for promotion of Th17 inflammation	[59]
	Lys48	ubiquitination	regulation of RORγt protein stability for inhibition of Th17 inflammation	[60]
	Lys69/Lys81/Lys99/Lys112	acetylation	regulation of RORγt binding ability for inhibition of Th17 inflammation	[63]
	Lys81	acetylation	regulation of IL-17-mediated inflammation	[64]
STAT3	Tyr705 and Ser727	phosphorylation	regulation of STAT3 activation	[65]
	Thr717	O-GlcNAcylation	inhibition of IL-10 production for promotion of colonic inflammation	[66]
IRF4		phosphorylation	promotion of Th17 inflammation	[71]
	Lys349	SUMOylation	regulation of IRF4 protein stability in Treg for inhibition of inflammation	[73]
c-Maf		phosphorylation	regulation of c-Maf binding ability for promotion of IL-21-mediated inflammation	[81,82]
	Lys33	SUMOylation	regulation of c-Maf binding ability for inhibition of IL-21-mediated inflammation	[78,83,84]
	Lys331/Lys345	ubiquitination	regulation of c-Maf protein stability	[86]
	Lys85/Lys297	ubiquitination	regulation of c-Maf protein stability	[87]
Blimp-1	Lys816	SUMOylation	regulation of Blimp-1 transcriptional activity and association with colonic inflammation	[94,95]
Foxp3	Ser19/Thr175	phosphorylation	regulation of Foxp3 transcriptional activity for inhibition of inflammation	[96,97]
	Ser422	phosphorylation	inhibition of Foxp3 binding ability for promotion of inflammation	[98]
	Ser418	phosphorylation	regulation of Treg population	[100,101]
	Lys31/Lys262/Lys267	acetylation	modulation of Treg function for inhibition of inflammation	[104]
		ubiquitination	regulation of Foxp3 protein stability for inhibition of inflammation	[105,106,107]
		O-GlcNAcylation	regulation of Treg cell lineage stability for inhibition of inflammation	[108,110]
